# Modification by Ubiquitin-Like Proteins: Significance in Apoptosis and Autophagy Pathways

**DOI:** 10.3390/ijms130911804

**Published:** 2012-09-19

**Authors:** Umar-Faruq Cajee, Rodney Hull, Monde Ntwasa

**Affiliations:** School of Molecular & Cell Biology, Gatehouse 512, University of the Witwatersrand, Johannesburg, 2050, South Africa; E-Mails: umar.cajee@students.wits.ac.za (U.-F.C.); rodney.hull@students.wits.ac.za (R.H.)

**Keywords:** ubiquitin-like, autophagy, apoptosis, immune response, DWNN, SNAMA, p53, Ubls, ubiquitin-proteasome, cancer

## Abstract

Ubiquitin-like proteins (Ubls) confer diverse functions on their target proteins. The modified proteins are involved in various biological processes, including DNA replication, signal transduction, cell cycle control, embryogenesis, cytoskeletal regulation, metabolism, stress response, homeostasis and mRNA processing. Modifiers such as SUMO, ATG12, ISG15, FAT10, URM1, and UFM have been shown to modify proteins thus conferring functions related to programmed cell death, autophagy and regulation of the immune system. Putative modifiers such as Domain With No Name (DWNN) have been identified in recent times but not fully characterized. In this review, we focus on cellular processes involving human Ubls and their targets. We review current progress in targeting these modifiers for drug design strategies.

## 1. Introduction

Ubiquitin is a 76 amino acid protein, which is covalently attached to a lysine residue on a target molecule via a conserved carboxy-terminal glycine residue. Ubiquitin consists of two α-helices and five β-sheets in a ββαββαβ arrangement. This structural fold (known as the ubiquitin globular β-grasp fold) is conserved in other proteins with no obvious sequence identity. These proteins are known as ubiquitin-like (Ubl) modifiers and their identification has been increasing steadily over the years leading to the characterization of an entire superfamily [1]. Although Ubls do not share high sequence similarity with ubiquitin, all members of this superfamily are characterized by the same three-dimensional structure. Initially, proteins that are tagged by ubiquitination were thought to be marked for degradation via the proteasome pathway but other roles of ubiquitination have now been identified. Similarly, there are emerging Ubls that are involved in cellular processes other than degradation of proteins. The ubiquitin module or ubiquitin-like (Ubl) modifiers are ligated to target proteins by three enzymatic steps involving an activating enzyme (E1), a carrier or conjugating enzyme (E2) and a ligase (E3) [2]. Key residues on the modifier are required for these enzymatic reactions and are conserved in the ubiquitin superfamily. At the *C*-terminal end of ubiquitin and most Ubls there are conserved di-glycine residues. The *C*-terminal glycine is the key residue required for an isopeptide bond often with a lysine on the substrate protein. Ubiquitin has a set of highly conserved lysine residues namely; K^6^, K^29^, K^48^ and K^63^ all of which can either be mono- or polyubiquitinated. However, other amino acids such as cysteine and the α-NH_2_ groups of the *N*-terminal residues of polypeptides can also act as sites for modification [3–5].

Ubiquitin-mediated degradation of proteins is employed in many cellular processes and is implicated in biological phenomena such as immune response, development and programmed cell death [6]. Here, we review human Ubls. We focus on their impact on apoptosis, autophagy and on immune response. Furthermore, we explore the evolutionary history and function of a new modifier known as DWNN (Domain With No Name) whose functional significance is still unclear.

## 2. Ubiquitin-Like Modifiers as Independent Modules

**Figure 1:**
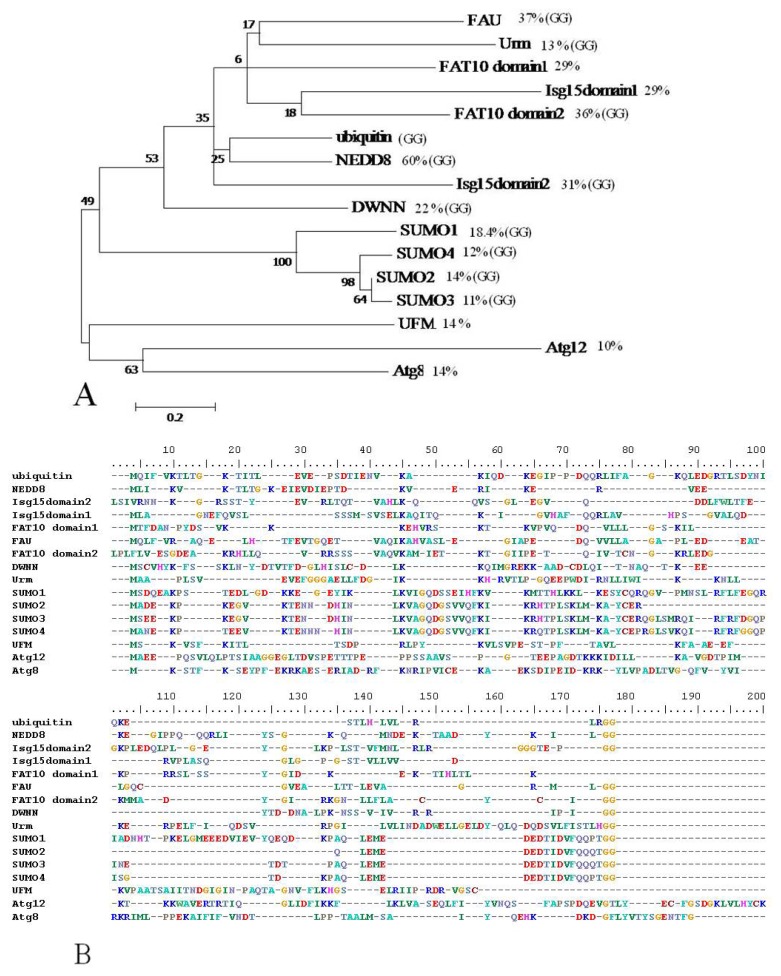
Phylogenetic analysis of ubiquitin-like modifiers and their protein sequence alignment. Protein sequences of the Ubl ubiquitin domains were initially aligned in MEGA 5, using the default ClustalW algorithm. The multiple sequence alignment was then analyzed and tweaked manually to ensure that important conserved residues (lysines, diglycine motif) were accurately aligned. The percentage similarity between each modifier and ubiquitin and the presence of a *C*-terminal di-glycine are marked on the phylogenetic tree (**A**). DWNN belongs to the group with proteins that have higher sequence identity with ubiquitin but it is the most distantly related in this group. The more distantly related UFM, Atg8 and Atg12 lack the conserved diglycine.

There are nine phylogenetically distinct classes of Ubls including SUMO, NEDD8, ISG15, FUB1, FAT10, Atg8, Atg12, Urm1, and Ufm1. We report here the existence of a putative modifier known as DWNN whose characteristics resemble those of Ubls but with poorly defined biochemical and functional features (Table 1). Ubls are involved in cellular activities such as apoptosis, autophagy and signaling pathways that mediate biological processes like cell proliferation, immune response and development. Ubls share low primary sequence similarity but phylogenetic analysis shows some evolutionary relatedness and clustering albeit with weak nodes (Figure 1). The most common feature is their three-dimensional fold.

### 2.1. Small Ubiquitin-Related Modifier (SUMO)

The small ubiquitin-related modifier (SUMO) was discovered serendipitously in 1996 when it was observed to stably modify RanGAP1 [20,21]. SUMO shares only 18% amino acid identity with ubiquitin but their three-dimensional structures are virtually super-imposable. However, it differs from ubiquitin in overall charge topology indicating differences in the nature of their interacting partners [22]. It is now regarded as the most studied posttranslational modification by a Ubl and seems to affect the widest range of proteins when compared to other Ubls [23,24]. Four functional SUMO genes have been identified in the human genome generating four isoforms (SUMO-1/2/3 and 4). The SUMO-3 gene was derived from SUMO-2 and the encoded proteins share 86% sequence identity. On the other hand, SUMO-1 shares only 44% sequence identity with SUMO-2 and −3 [25]. SUMO-4, encoded by a separate gene (SUMO-4), shares 85% identity with SUMO-2 and is expressed mainly in the kidney [9].

SUMO is conjugated via the *C*-terminal glycine to an ɛ-amino group on an internal lysine residue of the target protein. SUMOylation occurs via a series of reactions catalyzed by SUMO-specific enzymes using a stepwise mechanism that is analogous to modification by ubiquitin [21,23]. All the SUMO genes encode a precursor with a short peptide beyond the *C*-terminal diglycine, but SUMO cannot be transferred to the target protein unless the *C*-terminal glycine is exposed. This is accomplished by desumoylating enzymes such as sentrin specific proteases. These enzymes serve three purposes; to process precursor SUMO to mature forms, to deconjugate SUMOylated proteins and to depolymerize SUMO chains [26]. The SUMOylation pathway begins with the ATP-dependent activation of SUMO at the *C*-terminus by a heterodimeric SUMO activation enzyme consisting of SAEI (Aos1)/SAE2 (Uba2) [27]. The activated SUMO is then transferred through a transesterification reaction to Ubc9, the only known SUMO-conjugating enzyme (E2), forming a SUMO-Ubc9 thioester intermediate. The specificity for the substrate is determined by both Ubc9 and the E3 ligase. Although not mandatory, SUMOylation usually occurs within a consensus motif ψKxE (where ψ is a large hydrophobic residue and x any residue). *In vitro* SUMOylation can occur without the presence of E3 ligases, regardless of the consensus motif. *In vivo*, E3 ligases often enhance the modification by catalyzing the transfer of SUMO from Ubc9 to the target proteins. This consensus sequence is enough to promote SUMOylation *in vitro* but *in vivo* conjugation mainly occurs when the substrate protein is located in the nucleus [28]. An exception to this is Rac1, a Rho-like GTPase that induces the cytoskeletal rearrangements required for cell migration. Increased Rac1 activity requires the E3 ligase PIAS3 to SUMOylate Rac1, leading to higher levels of Rac1-GTP. This modification occurs in the cytoplasm and serves to increase cell migration and invasion [29].

As evidence shows in this section, SUMOylation confers several functions on target proteins namely; protein stability, subcellular localization, transcription activation, DNA repair, and other cellular events [21,23,30]. Modification of IκBα by SUMO prevents its degradation by ubiquitination thus maintaining a stable NF–κB in the cytoplasm. NF-κB, a transcription factor that is induced during cellular activities such as inflammatory response, is sequestered in the cytoplasm by IκB inhibitors in unstimulated cells. Signal induced activation of NF-κB is mediated by the ubiquitination and subsequent proteasomal degradation of IκBα. SUMO inhibits this degradation by modifying IκBα at K^12^ and K^22^; the same sites required for ubiquitination [31,32]. Thus SUMO regulates this signaling pathway by competing with ubiquitin thereby antagonizing the degradation of NF-κB.

SUMOylation is involved in both negative and positive regulation of gene transcription. SUMO does not bind directly to DNA but appears to influence transcriptional activity indirectly by interacting with transcription factors as mutations in SUMO modification sites on transcription factors such as Elk-1, Sp-3, C/EBPs and c-myb result in repression [24]. Regulation of transcription repression by SUMO is illustrated by SUMOylated histone deacetylases (HDACs). Point mutations on SUMOylation sites of class I and II HDACs cause defects in their ability to repress transcription. SUMOylation of these proteins is coupled to nuclear localization and requires the presence of an intact nuclear localization signal. Furthermore, it occurs at the nuclear pore complex and is catalyzed by a SUMO E3 ligase, the nuclear pore complex (NPC) RanBP2 protein [33].

In the Wnt signaling pathway SUMO is reported to have a positive effect on gene transcription. A point mutation in TCF (Tcf-4^K297R^) results in reduced activation by β-catenin and the SUMO E3 ligase PIASy compared to wild type Tcf-4. In other experiments, it was shown that the knock down of the desumoylation enzyme, Axam increases SUMOylation and activation of Tcf-4 [34,35]. SUMOylation also converts the heat shock transcription factor, HSF1 into a DNA-binding form thus promoting transcription in response to stress such as elevated temperature [36].

SUMOylation affects post-replication DNA repair by influencing several molecules involved in the cell cycle or in replication. The p53 family of proteins (including p63 and p73) which are regarded as guardians of the genome are regulated by SUMOylation at their *C*-termini. The prototypical tumor suppressor protein p53 is known to be constitutively and negatively regulated by the ubiquitin ligase Mouse double mutant 2 (Mdm2) in normal cells. SUMOylation of p53, usually at K^389^, increases after its DNA damage induced stabilization [37]. Moreover, over-expression of SUMO-1 activates wild type p53 transcriptional activity but not that of the mutant p53^K386R^. This points to the involvement of SUMOylation in p53 DNA damage repair activities. It is noteworthy that ubiquitin and SUMO-1 do not compete for the same site on p53 in this instance [38]. The proliferating cell nuclear antigen (PCNA), a DNA-polymerase sliding clamp involved in replication and DNA repair, is conjugated to SUMO-1 by the SUMO conjugating enzyme Ubc9 after increased DNA damage. Interestingly, PCNA is SUMOylated and ubiquitinated on the same residue; (K^164^) [39]. In an extensive review, [40] discuss a wide range of prominent nuclear proteins that are modified by SUMO and note that all the major DNA-repair pathways are regulated by either ubiquitination, SUMOylation or both.

Finally, the last to be identified SUMO-4 appears to have a different functional mechanism compared to the others. In its mature form it has a proline residue at the *C*-terminus instead of the conserved glycine. This introduces conformational constraints limiting the ability of SUMO to be part an *H*-bond network required in covalent interactions. SUMO-4 was predicted therefore to participate in non-covalent interactions and was shown to function in conjugating DNA repair proteins functioning in base excision repair [41]. In a separate study a M55V polymorphism in SUMO-4 was found to be associated with type 1 diabetes mellitus [9]. This may be consistent with the high expression of SUMO-4 in kidneys.

### 2.2. Neural Precursor Cell-Expressed Developmentally Down-Regulated (NEDD8)

The neural precursor cell-expressed developmentally down-regulated (NEDD8) is another highly studied ubiquitin-like protein and shares the highest sequence identity (approximately 60%) with ubiquitin [42]. NEDD8 modifies target proteins in a manner analogous to ubiquitin—in a series of reactions involving NEDD8 activating E1-like enzyme (APP-BP1/Uba3), NEDD8-specific E2 enzymes (Ube2F or Ubc12) and the really interesting new gene (RING)-finger protein ROC1 (NEDD8 E3 ligase). The nature of chain formation by NEDD8 has been investigated *in vitro* and found to have subtle differences to polyubiquitination. NEDD8 chains can be linked via the catalytic cysteine residue of Ubc12 (E2) forming a thioester bond in the absence of ROC1 (E3) activity. Indeed ROC1 ligase E3-inactive mutants enhance poly-neddylation and a RING finger inhibitor enhances poly-neddylation of Ubc12 [43]. In yeast, NEDD8 was found to be ligated to members of the Cullin/Cdc53 family thus functioning as part of the Skp1-Cdc53/Cul-1-F-box (SCF) complex [44]. All human Cul family proteins were shown to be targets of Neddylation and NEDD8 and Cul family protein tissue distribution coincides [45]. Neddylation therefore is important for a variety of biological processes and has implications for pathological conditions especially those related to proliferation of cells such as cancer.

Neddylation is required for the regulation of the multifunctional transcription factor—NF-κB, which is crucial in immune response and apoptotic pathways. In the NF-κB pathway scenario, NEDD8 promotes the function of the SCF E3 ligase by recruiting the ubiquitin conjugating enzyme E2 thus augmenting the function of the SCF complex as a E3 ligase for ubiquitination of IκBα [46]. Cullins serve as scaffold proteins for the assembly of multicomponent Cullin RING E3 ligases (CRLs). These CRLs participate in the ubiquitination and proteasomal degradation of target proteins. In addition to this, there is a large number of substrate recognition subunits that assemble onto the Cullin scaffold allowing for a wide variety of targets to be ubiquitinated [47,48]. CRLs target numerous substrates and, therefore, regulate a wide range of biological processes. These include cell growth, development, signal transduction, transcriptional control, genomic integrity and tumor suppression [49]. Mutations in *CUL7* and *CUL4B* genes are linked to hereditary human diseases [48]. Cullin4 mutant mice exhibit increased resistance to UV induced carcinogenesis as a result of an impairment in the nucleotide excision repair pathway underlining the important role played by Cullins in DNA damage repair. This is accomplished through the selective degradation of damage sensors such as the DDB2 (damage-specific DNA-binding protein) and XPC (*Xeroderma pigmentosum*) and checkpoint effectors p21/CIP1/WAF1 [50]. Nearly all members of the Cullin family require the mononeddylation of a conserved lysine residue to stimulate the ubiquitin activity of the CRLs [48]. Neddylation of Cullin3 leads to the negative regulation of the hedgehog pathway, which has been implicated in cancer [51]. Evidence obtained from deletion studies using *C. elegans*, *D. melanogaster* and *M. musculus* reveal that Cullins affect many physiological processes to do with cell proliferation and survival. Consequently, deletion of genes that encode Cullins has an impact on biological processes and phenomena like development and apoptosis [49].

In addition to Cullins, the most studied substrates for neddylation are other proteins involved in cell proliferation, viability and development. These include p53, EGFR, pVHL, ribosomal proteins and BRCA (breast cancer-associated protein) [52,53]. The identification of ribosomal proteins was conducted in a proteomics study revealing a subset of large and small ribosomal proteins that are neddylated. It was also shown that loss of neddylation causes protein instability [54].

NEDD8 is involved in the regulation of gene transcription by enhancing DNMT3b-dependent DNA methylation. It is well known that in cancer cells many promoters become aberrantly methylated. DNA methyltransferases such as DNMT3 interact with NEDD8 modified proteins (DNMT3b) and directly with NEDD8 (DNMT3a). DNMT3 is also known to interact with neddylated Cullins resulting in the formation of repressive chromatin marks [55].

Lastly, neddylation influences the IAP (inhibitor of apoptosis) pathway in both vertebrates and invertebrates. This was demonstrated by the fact that knocking down the expression of isopeptidase enzymes that remove the NEDD8 modification, leads to suppression of *Drosophila* Hid- and Reaper- induced apoptosis. Neddylation contributes to the regulation of apoptosis by controlling the ability of IAPs to mediate the conjugation of NEDD8 to effector caspases. Furthermore, downregulation of Deneddylase (DEN1), an enzyme that is responsible for the removal of NEDD8 from target proteins, was found to suppress apoptosis further supporting that neddylation prevents apoptosis [56]. Overall, the pivotal role neddylation plays in replication and cell cycle events points to its importance in cell proliferation, death and survival.

### 2.3. Human HLA-F Adjacent Transcript 10 (FAT10)

FAT10 is an 18 kDa Ubl, which shares moderate sequence similarity with ubiquitin, (29% and 36% at the *N*- and *C*- termini respectively). It was first identified amongst genes in the HLA-F locus and found to be expressed in lymphoid cell lines [57]. Soon after that, FAT10 was shown to be constitutively expressed in lymphoblastoid cells and in dendritic cells and to be induced in certain other cells by pro-inflammatory stimuli [58]. These two studies reveal the unique features of this modification, namely that it is found in vertebrates only and is expressed by specific stimuli in a tissue-specific manner. Also known as diubiquitin (owing to the two ubiquitin-like domains in tandem, head-to-tail) this Ubl relies on its *C*-terminal diglycine motif for modification of its substrates [1]. Both domains contain a lysine residue mapped to ubiquitin K^48^ which could serve as the site for FATylation [59]. FAT10 modification is mediated through the Uba6 (E1) and USE1 (E2) enzymes, which are specific to both FAT10 and ubiquitin [60,61].

FAT10 has potential function in regulating the cell cycle, tumorigenesis, inhibition of cell proliferation and in survival. It also plays a significant role in immune response. For example, FAT10 expression is induced by interferon-γ and tissue necrosis factor α (TNFα) [62]. Deletion of mouse FAT10, which is highly similar to the human one, results in lymphocytes that are prone to spontaneous apoptotic death and sensitive to endotoxin exposure [63]. Furthermore, FAT10 appears to mediate the activation of NFκB, a key mediator of innate immunity [64]. FAT10 has been shown to change the conformation of p53, thereby stimulating its transcriptional activity [65]. It is also implicated in the regulation of mitosis and chromosomal stability [66] and in caspase-dependent apoptosis [67]. Indeed, increased FAT10 gene expression has been observed in several oncogenic conditions including liver, gynecological and gastrointestinal cancers [59,67]. Notably, FAT10 is overexpressed when cancer occurs in a pro-inflammatory environment consisting of interferon-γ and tumor necrosis factor α-cytokines that synergistically upregulate the expression of this modifier [62].

FAT10 has a short half-life due to rapid degradation by the proteasome in a process that seems to be dependent on polyubiquitination, as degradation is inhibited in cells that express a mutant form of ubiquitin that lacks the conserved lysine residue required for polyubiquitination [68].

It has been found that in Hep3B, a human cell line derived from the liver, FAT10 is repressed when the cells express p53. Furthermore, p53 was also shown to bind to FAT10 promoter in *in vivo* studies [69]. FAT10 is therefore transcriptionally controlled by p53.

### 2.4. Interferon Stimulated Gene 15 (*ISG15*)

The ubiquitin-like ISG15 protein is a 17 kDa protein that is understood to be primarily an anti-viral response gene whose expression is induced by type I IFN (interferon), LPS (lipopolysaccharide), and pI:pC, a synthetic inosine polymer that resembles the RNA of infectious viruses [70]. ISG15 is one of the various proteins induced by interferon (IFN)α/β and also amongst the first Ubls identified [71]. Upon discovery, ISG15 was found to be under the control of an upstream enhancer element that was IFN inducible [72]. This enhancer element located in the ISG15 promoter includes a putative p53-responsive element. The induction of ISG15 by p53 was demonstrated in HeLa cells using a temperature sensitive mutant of p53. Transfected cells grown at 37 °C expressed a mutant form of p53 that was unable to induce ISG15 expression. When the temperature was lowered to 32 °C allowing p53 to resume the wild type form, ISG15 was induced within 6 h. Interestingly, dsRNA induced ISG15 in a p53-dependent manner whereas induction by virus infection was not dependent on p53 suggesting that dsRNA and p53 may have overlapping profiles of induced genes and employed different signaling pathways [73].

ISG15 has so far been found in higher eukaryotes only and is absent from insects implying that ISG15 is restricted to animals that have evolved an IFN signaling pathway [74]. Like ubiquitination, conjugation of substrate proteins by ISG15 (ISGylation) follows a three-step enzymatic cascade using an E1 activating enzyme (Ube1L), an E2 conjugating enzyme (UbcH6 and UbcH8), and several E3 ligases such as EFP (estrogen-responsive finger protein) and the HECT (homologous to E6-AP *C*-terminus)-type E3 ligase Herc5. Both E3 ligases are inducible by interferon [75,76]. Ube1L is a 112 kDa protein that is a specific ISG15-activating E1 enzyme. In addition, Ube1L has a *C*-terminal ubiquitin fold domain that is required for the transfer of ISG15 [77]. UbcH8 and UbcH6 are ISG15-conjugating E2 enzymes that are induced by type I IFNs and viral infection. However, UbcH8 was originally identified as an ubiquitin-conjugating E2 enzyme interacting with other ubiquitin E3 ligases, such as Parkin and Dorfin [77]. Herc5 is the predominant ligase for ISG15, as knock down of the encoding gene by RNA interference abolishes most IFN-induced ISGylation [78].

ISG15 has characteristics pointing to its vital role in the innate immune response to viral infections. Over 150 to 300 proteins have been identified as ISGylation targets and found to be newly synthesized proteins associated with polyribosomes [11,79]. Mass spectroscopic identification of ISGylated proteins also revealed a set of IFN-induced and Jak/STAT pathway signaling molecules [74]. EFP is a member of the TRIM family of proteins which are implicated in antiviral responses [80]. Moreover, the expression of EFP in human culture cells was found to be stimulated by the binding of STAT1 to an interferon–stimulated response element (ISRE) in an interferon dependent manner [81].

The involvement of ISGylation in antiviral response is now well documented and its impact on specific viruses has been recorded. The over-expression of ISG15 in cell culture suppresses the replication of a broad range of viruses including Sindbus virus [82,83], HIV [84], Ebola VP40 [85] and Herpes [83]. In addition to this, the influenza NS1B protein antagonizes ISGylation [86] while at the same time it is a target for Herc5-dependent ISGylation [87]. Infection by NS1A inhibits host cell pre-mRNA processing, resulting in the inhibition of IFN function but ISGylation of NS1A impairs the ability of the virus to replicate [78]. Consequently viruses have evolved specific proteins that function to deconjugate ISG15 from target viral proteins and thereby evading the anti-viral response [74].

The human pathogen Crimean Congo hemorrhagic fever virus (CCHFV) evades the innate immune system by deubiquitinating components of the antiviral signaling pathways. Some of the proteins in this pathway rely on the modification of proteins with the Ubl modifier, ISG15. The virus can shut down this pathway through the viral encoded ovarian tumor (vOTU) family deubiquitinase, which is able to target ISG15 modifications. Despite differences between ISGylation and ubiquitination enzyme kinetics, vOTU family deubiquitinase cleaves ubiquitin and ISG15 with similar kinetics. This is most likely due to the similarities in the *C*-terminal ubiquitin-like fold of ISG15 (ISG15-C). However, there is a second unique binding site for vOTU in ISG15-C [88].

The viral response via the JNK pathway is also regulated by ISG15. When ISGylation is extended by the deletion of the ISG15-specific protease UBP43, IFN signaling results in extended STAT1 activation and INF-mediated gene activation [89]. IFN stimulates the binding of MAPKs to filamin B, stimulating JNK activation and resulting in JNK-mediated apoptosis. ISGylation of filamin B prevents the binding of these MAPKs and prevents JNK mediated apoptosis [90].

### 2.5. Autophagy-Related (ATG) Genes

Autophagy is a process of self-degradation whereby cytoplasmic components are sequestered within double-membrane autophagosomes and subsequently delivered to the lysosome where they are broken down into individual amino acids. Autophagy is important in diseases such as neurodegeneration and cancer [91]. Two Atg proteins, Atg8 and Atg12 have been identified as Ubls that possess a ubiquitin-like fold and adopt a ubiquitin-like mechanism to modify substrate proteins [92].

Atg8 is a lipid-conjugated ubiquitin-like protein. Atg activation is catalyzed by Atg7 (E1) forming a thioester bond between its own C^507^ and the *C-* terminal glycine of Atg8. Atg8 is then transferred to the E2 enzyme Atg3. Finally, the Atg12-Atg5-Atg16 complex acts as an E3 ligase transferring Atg8 to phosphatidylethanolamine via a *C-* terminal glycine [93]. The transfer of Atg8 to lipids is initiated by the removal of a carboxy-terminal arginine residue by the cysteine protease Atg4 revealing a carboxy-terminal glycine [94].

The enzyme cascade responsible for the transfer of Atg12 to its target molecules involves other Atg proteins Atg5, Atg7 and Atg10. Atg7 functions as the E1 enzyme and Atg10 as an E2 [1,95]. Atg7 shows little sequence similarity to other E1 enzymes, however, it shares a conserved ATP binding domain: a metal-binding motif and an active-site cysteine residue with other E1 enzymes including Uba2, Uba3, Uba4 and Uba5. The ATP-binding domain is essential for the formation of the Atg12-Atg5 conjugate [16]. The E2 enzyme Atg10 forms a thioester bond with activated Atg12 on C^133^. Apg10p, a protein conjugating enzyme in yeast, shows no significant similarity to other E2 enzymes [96]. Atg12 is then transferred to Atg5. There is no identified typical E3 enzyme involved in this transfer. The Atg12-Atg5 complex interacts further with a small coiled-coil protein, Atg16, and Atg12-Atg5-Atg16 forms a multimeric complex through the homo-oligomerization of Atg16 [97]. This multimeric complex is found on the outer side of the autophagosomal membrane and acts as an E3 ligase in the transfer of Atg8 [77]. Atg3 has also been identified as a substrate for Atg12. Atg3 is an E2 enzyme and performs this function by autocatalytically transferring Atg12 to itself. The Atg12-Atg3 complex formation limits the increase in mitochondrial mass preventing mitochondrial pathways from rescuing dying cells and, thereby, promoting apoptosis [98]. Although the complex dissociates once the assembly of the phagosome is complete, no specific Atg12 hydrolase has been identified [77]. The mechanism by which this process occurs is therefore not clear.

### 2.6. Ubiquitin-Related Modifier 1 (Urm1)

Urm1, first identified in yeast together with its E1 enzyme Uba4, was found to have the unique properties of affinity for prokaryotic enzyme systems and resemblance to prokaryotic proteins involved in sulfur transfer [99]. It was, however, found to have a β-grasp structure similar to ubiquitin [100]. Urm1 is conserved across all eukaryotes and shares structural similarities with evolutionarily ancient sulfur carrier proteins. It is an unusual ubiquitin-like modifier as it contains a thiocarboxylate group at the *C*-terminus and is involved in the transfer of a sulfur group and modification of certain tRNA species [77]. It has been found that reduction of cellular Urm1 levels causes severe cytokinesis defects in HeLa cells, resulting in the accumulation of enlarged multinucleated cells [19]. The unique features of this modification may represent some evidence for the ubiquitin system in prokaryotes.

### 2.7. Ubiquitin-Fold Modifier 1 (Ufm1)

Ufm1 is a 9.1 kDa protein, sharing 16% sequence identity with ubiquitin [101]. Unlike other Ubls Ufm has only a single glycine at its *C-*terminus [101]. The newly synthesized protein is cleaved by UfSP1/2 to expose this glycine, forming a mature Ufm1 [101]. Activation by the E1 Uba5 is followed by transfer to Ufc1 (E2) [102]. Recently, a Ufm1 E3 ligase and substrate have been identified and named Ufl1 and C20orf116, respectively [102]. This protein conjugate is then cleaved by the UfSPs mentioned earlier. Although Ufm itself is not yet fully understood regarding function, the conjugation was found to be abundant in the liver and lungs of Ufm1-transgenic mice [102]. The E1, Uba5, is essential for erythroid differentiation in mice, and deficiencies cause death from anemia, as a result of impaired differentiation as well as an increase in apoptosis of megakaryocyte and erythroid precursors [103]. This is the newest form of modification, and information about its biological significance is still patchy.

### 2.8. Domain with no Name (DWNN)

**Figure 2:**
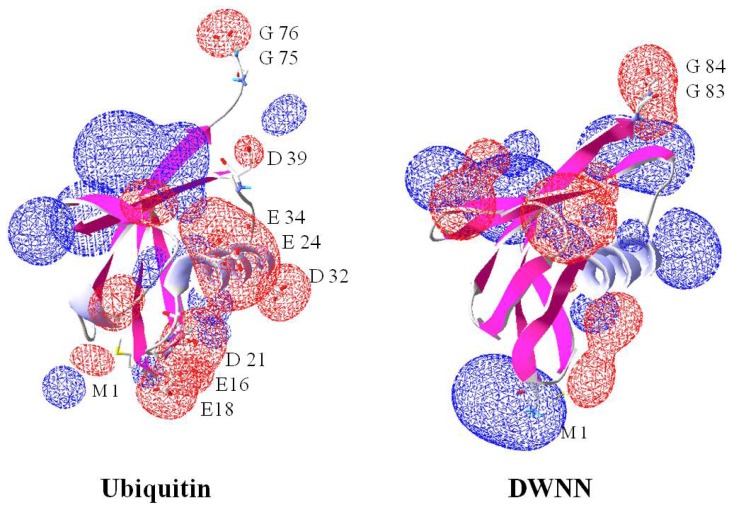
Comparison of charge topologies of DWNN and ubiquitin viewed in SPDBviewer. The positive charges are shown in blue while the negative charge is shown in red. Ubiquitin shows an equal charge distribution while DWNN shows a much higher positive charge. This implies that ubiquitin will probably be able to associate with a wider range of proteins due to it having negative and positive areas. Note the diglycine gives a negative charge in both molecules allowing them to associate with the positively charged lysine during modification.

The recently discovered putative ubiquitin-like modifier DWNN shares about 28% identity with ubiquitin, but together they possess an almost superimposable three-dimensional structure. DWNN is a 76 residue protein found in vertebrates as an *N*-terminal domain of the Retinoblastoma Binding Protein 6 (RBBP6) family (also known as mouse proliferation potential proteins (P2P-R) or p53-associated cellular protein-testes derived (PACT)) or as an independent module encoded by its own transcript. It lacks the conserved K^48^ and K^63^, but in most cases K^6^ and K^29^ are conserved [18,104,105]. The residues are not conserved in protists and worms. Although the three-dimensional structure of DWNN is similar to that of ubiquitin the charge topologies are significantly different (Figure 2). Ubiquitin has a much more prominent negative surface compared to DWNN whose surface is largely positively charged. These features suggest that the two domains have different interacting partners. This domain is not found in prokaryotes but is present in all eukaryotes. Similarly, the RBBP6 protein family is found in eukaryotes only and appears to exist as a single copy gene with no transcript variants in plants and in invertebrates.

**Figure 3:**
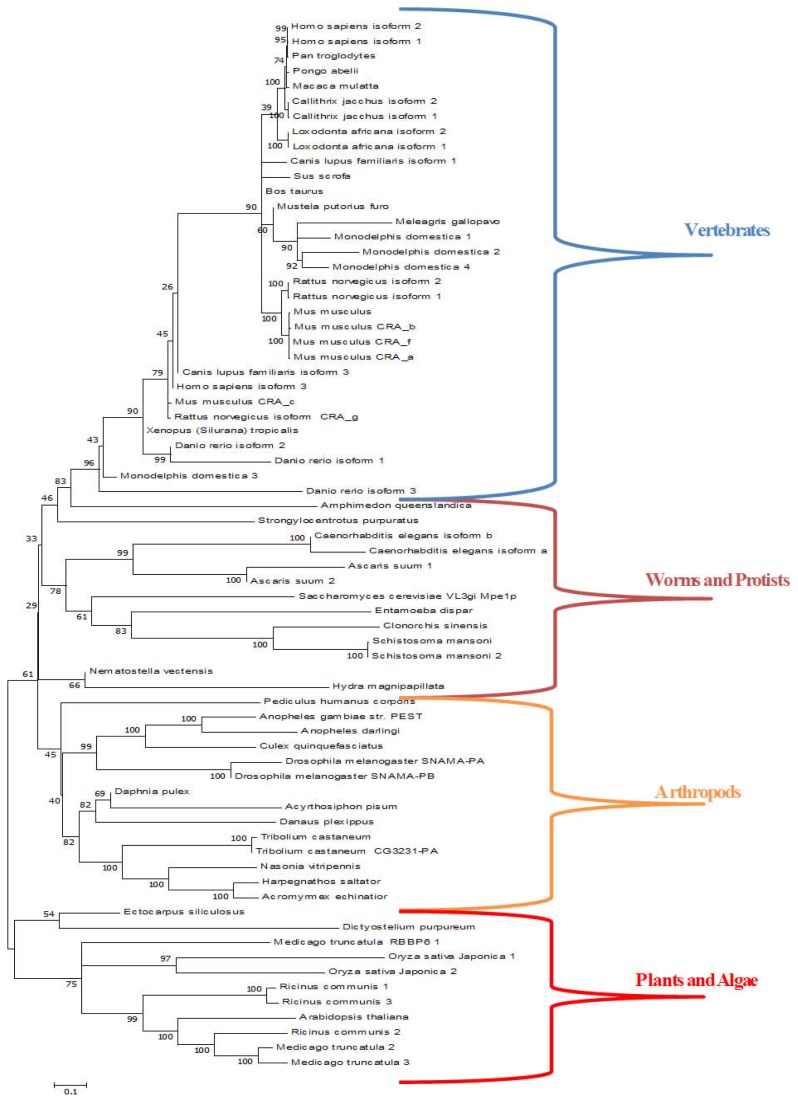
Phylogenetic analysis of proteins with DWNN domains. Full RBBP6 orthologous protein sequences were aligned in MEGA 5, using the default ClustalW algorithm. The multiple sequence alignment was then analyzed and tweaked manually to ensure important conserved residues (lysines, di-glycine motif) were accurately aligned.

In plants, invertebrates and vertebrates RBBP6 proteins have a multi-domain arrangement with combinations of the p53- and Rb-binding, and SR domains in addition to the core ubiquitin-like domain that is linked to a zinc knuckle and RING finger (Figure 3) [104,105]. With the exception of vertebrate orthologues, DWNN lacks the characteristic *C*-terminal diglycine motif required for conjugation of ubiquitin and Ubls to substrates. The significance of this is not yet clear. These proteins are associated with cell- and tissue-specific cell proliferation, apoptosis and mRNA splicing. In some experiments using mice RBBP6 proteins have been shown to enhance the activity of Mdm2 (mouse double mutant). Interestingly, *Rbbp6* and *mdm2* mice also have a similar phenotype. The loss of RBBP6 has an embryonic lethal phenotype while double mutants of *Rbbp6* and *p53* develop better [106]. This phenotype is similar to that of *mdm2^−/−^/p53^−/−^* [107]. Taken together, these observations point to a close association between the function of Mdm2 and RBBP6. Although DWNN was identified earlier than UFM1, very little is known about the enzymology of conjugation. Indeed it is not known whether this domain can be conjugated to other proteins in the ubiquitin-like fashion. The existence of the mammalian DWNN domain as an independent module, however, points to this possibility.

## 3. Proteins with an Ubiquitin Domain

There is a set of cellular multi-domain proteins with Ubls that are often arranged together with the RING-finger motif. These proteins are encoded by a single transcript and consist of the ubiquitin-like domain usually at the *N*-terminus and a RING-finger motif somewhere along the length of the protein. In this section we discuss the structural organization and function of the currently characterized proteins (Table 2).

### 3.1. Homocysteine-Inducible, Endoplasmic Reticulum Stress-Inducible, Ubiquitin-Like Domain Member 1 (Herpud1)

Herpud is a 55 kDa protein first identified as playing a role in endoplasmic reticulum (ER) stress response during hyperhomocysteinemia [115]. The *N-*terminus of Herpud has a ubiquitin-like domain which is likely to facilitate Herpud degradation by the ubiquitin proteasome system [115,117]. Herp is localized in the ER of neurons and other tissues and may act in ER-folding and in ER-associated degradation (ERAD) of proteins [118]. So far Herpud function is associated with neuroprotection through mitochondrial and Ca^2+^ homeostasis in the brain [119] and prevention of apoptosis in response to stress [119,120]. Herpud may also decrease protein load and increase the capacity the ER to fold [120].

### 3.2. Parkin

The human Parkin gene was found to be responsible for the pathogenesis of autosomal recessive juvenile parkinsonism. It is a 51.6 kDa protein with a ubiquitin-like domain at the *N-*terminus and two RING finger motifs separated by an in-between-RING-finger (IBR) motif at the *C*-terminus [121,122]. It has been demonstrated that Parkin functions as an E3 ligase and that it interacts with a number of proteins including E2 enzymes and putative substrates [123,124]. A number of these Parkin substrates are proteins that are linked to neurodegenerative disorders. Furthermore, the *Drosophila* homolog of the *parkin* gene is an E3 ligase that interacts with Ubc-H8 and it appears to act via the RING-finger motif. Parkin also appears to act in a pathway of p53-mediated apoptosis [125].

### 3.3. Retinoblastoma Binding Protein 6 (RBBP6)

The Retinoblastoma Binding Protein 6 (RBBP6) proteins also known as Proliferation Potential related (P2P-R) or the p53-associated cellular protein-testes derived (PACT) in the mouse [126,127] or SNAMA in *Drosophila* are found in all eukaryotic organisms but not in prokaryotes. They are characterized by the DWNN domain at the *N*-terminus and an associated zinc knuckle and RING-finger motif. Orthologous forms have different combinations of domains and motifs including the p53 binding domain, RB-binding domain, the SR motif and others [18,104,105,126,128].

RBBP6 proteins are found as single isoforms in invertebrates and in the plant kingdom but various isoforms are predicted in vertebrates (see Figure 3). Null mutants of mouse PACT are not viable, but can be rescued by simultaneously knocking out p53 [106]. An alternatively spliced version of PACT is involved in cell cycle arrest and is responsible for camptothecin-induced apoptosis [128]. PACT itself most likely regulates p53 activity by interfering with the ability of p53 to bind DNA [127]. PACT also interacts with Mdm2, and enhances Mdm2-mediated ubiquitination and degradation of p53 suggesting that it is a negative regulator of p53 [106].

The *Drosophila* orthologue was found to be important for apoptosis and to play an active role in nucleic acid metabolism during embryonic development. *SNAMA* (*mini-me* locus) is expressed throughout development with higher levels during early embryonic development compared to later stages [18,104,105]. The RBBP6 proteins have not been shown to mediate E3 ligase activity even though they have the hallmarks of E3 ligase such as the RING-finger motif and the ubiquitin-like domain.

### 3.4. Ubiquilin

Ubiquilin is a protein consisting of three domains; a ubiquitin like domain, a ubiquitin associated domain and a third domain that is highly conserved in several other large ubiquitin-like proteins. Ubiquilin expression was originally found in the testes [129].

Yeast two-hybrid assays identified ubiquilin as a protein that interacts with protein disulfide isomerase (PDI). Following cellular stress such as hypoxia both these proteins localize to the endoplasmic reticulum. Hypoxia causes the accumulation of immature proteins, which may eventually initiate cell death. Over-expression of ubiquilin leads to a decrease in hypoxia induced apoptosis. This protection is increased when PDI is over-expressed in conjunction with ubiquilin. As this only protected cells from hypoxia induced apoptosis it was deduced that ubiquilin selectively blocks ER stress induced apoptosis. Ubiquilin and PDI act by delaying the expression of DNA damage-inducible transcript 3 protein [130].

## 4. Potential for Therapeutic Intervention

The involvement of several ubiquitin-like modifiers in cancer pathogenesis was recently reviewed by [131]. Modification and de-modification by the Ubls and ubiquitin as well as poly- and mono-ubiquitination, alter the function of a number of proteins which show direct involvement in various cancer pathogeneses, including lymphomas, melanomas, lung, ovarian, prostrate and leukemia, among others. Specifically, SUMO activity in chromatin remodeling, through sumoylation of proteins, cofactors and transcription factors involved therein, directly influences transcriptional activity and regulates other signaling pathways. One such pathway is the Wnt pathway. When β-catenin is stimulated by the Wnt ligand it localizes to the nucleus to recruit chromatin remodeling complex and thereby activates transcription. Tumor metastasis is promoted by the expression of the *KAI1* gene, which is usually repressed by a SUMO modified Reptin (involved in β-catenin chromatin remodeling complex). Exploiting this interaction by downregulating Ubc9 (a Sumo E2), or by over-expressing SENP1 (a desumoylating enzyme) inhibits metastasis by promoting KAI1 expression [131].

ISG15 conjugation may be similarly implicated in lung cancer and APL (acute promyelocytic leukemia). Ube1L and Ubp43 levels are affected by these cancers. After treatment aimed at these cancers (doses of all transretinoic acid), Ube1L mRNA is increased. However, detailed effects of ISG15 and its enzymes in tumor suppression are yet to be explained [131].

The innate immune system plays a role in tumor suppression through the activation of IFN. In this way ISG15 plays a role in tumor suppression. Camptothecin and other chemotherapies can induce ISG15 expression in a JAK/STAT independent method. This implies a separate pathway to that used in an anti-viral response. Additionally camptothecin induced ISG15 activation results in a unique set of proteins being ISGylated [132]. When recombinant human IFN was added to colorectal cancer xenografts in combination with camptothecin, the result was an increase in the antiproliferative effects of camptothecin [133].

## 5. The Ubiquitin and Ubiquitin-Like Protein System as a Drug Target

It is clear that the ubiquitin-proteasome system is implicated in a number of physiological processes. Primarily, it controls the level or rather turnover of proteins in eukaryotic cells thus playing a key regulatory role in signaling events. After many years of concerted research, the ubiquitin-proteasome system has now been extensively studied making it possible to design drugs that target specific events during the protein degradation pathways (Table 3). Due to the involvement of this system in growth related cellular activities, new drugs based on it are under development to target cancer therapy [134]. The first drug in the class that functions by inhibiting the proteasome is bortezomib, a boronic acid dipeptide that went through clinical trials with promising results (Table 3). Bortezomib, a potent, selective, and reversible inhibitor of the proteasome provides prospects for the treatment of relapsed multiple myeloma that is refractory to conventional chemotherapy [135–137]. In 2008, bortezomib (trading as Velcade^®^) obtained accelerated approval by the United States Food and Drug Administration (FDA) for the treatment of patients with multiple myeloma by injection [138,139]. Since then improvements are being investigated. MLN4924 is the second compound in clinical trials that targets the NEDD8 system (Table 3).

The multi-step enzymatic pathway involved in conjugating the ubiquitin tag provides opportunities for finding “druggable targets” to develop treatment for cancer and other diseases. In this process specificity for substrates is determined mainly at the E3 ligase level and in the case of Ubls, at both the E2 and E3 level. E3 ligases belong to three major classes namely; *N*-end rule E3s, E3s containing the HECT (Homology to E6AP *C*-Terminus) domain, and E3s with the RING (Really Interesting New Gene) finger. The potential for E3 ligases as targets for cancer therapy is discussed extensively in a review by [140]. Recently, an inhibitor of Mdm2 known as nutlin entered preclinical studies and is being considered for use together with Velcade^®^ for the treatment of multiple myeloma [141]. Mdm2 is an E3 ligase of the RING-finger type and functions in normal cells by negatively controlling the pro-apoptotic tumor suppressor p53. Mdm2 promotes the ubiquitination of p53 thus targeting it for degradation by the proteasome system.

The Ubls discussed in this review provide new opportunities for drug design as they target specific substrates and biological processes such as cell-cycle regulation, proliferation, apoptosis and DNA repair. SUMO in particular stands out as an attractive target for drug discovery as it requires specific enzymes for conjugation and is implicated in many biological processes. Its role in carcinogenesis and neurodegenerative diseases is well documented [142]. Ubc9, the only E2 enzyme for SUMOylation, has already been proposed as a target for cancer therapy since it is over-expressed in some tumors such as ovarian carcinoma, melanoma and lung adenocarcinoma [143]. Arsenic trioxide has been found to increase the ubiquitination and subsequent degradation of promyelocytic leukemia (PML) by promoting its SUMOylation by SUMO-1 and −2 [144]. It is now regarded to have significant efficacy in treatment of relapsed patients with acute promyelocytic leukemia (APL) [145].

## Figures and Tables

**Table 1:** List of ubiquitin-like modifiers and their functions.

Modifier	Functions	Accession	Reference
SUMO-1	Nuclear transport, DNA replication and repair, mitosis and signal transduction	NP_001005781	[7,8]

SUMO-2	Nuclear transport, DNA replication and repair, mitosis and signal transduction	NP_001005849	[7]

SUMO-3	Nuclear transport, DNA replication and repair, mitosis and signal transduction	NP_008867	[7]

SUMO-4	May modulate protein sub-cellular localization, stability or activity. Upon oxidative stress, conjugates to various stress defense proteins. Negative regulation of NF-kappa-B-dependent transcription	NP_001002255	[9]

NEDD8	Cell cycle control and embryogenesis	NP_006147	[7]
	May be involved in the formation of aggresomes		[10]

ISG15	Modifies STAT1, SERPINA3G/SPI2A, JAK1, MAPK3/ERK1, PLCG1, EIF2AK2/PKR, MX1/MxA, and RIG-1. May serve as a trans-acting binding factor directing the association of ligated target proteins to intermediate filaments. May also be involved in autocrine, paracrine and endocrine mechanisms. Displays antiviral activity	NP_005092	[7,11]

FAT10	Protein degradation. Regulates TNF-alpha-induced and LPS-mediated activation of innate immunity. Mediates mitotic non-disjunction and chromosome instability, in cancers. May be involved in the formation of aggresomes when proteasome is saturated or impaired. Mediates apoptosis in a caspase-dependent manner	NP_006389	[1,12]

Ubl5	Negatively regulates p53	NP_001041706	[13,14]

FAU	Translation Gene expression. Viral infectious cycle. Endocrine pancreas development. Cellular protein metabolic process	NP_001988	[15]

Apg12/Atg12	Autophagic vacuole assembly. Negative regulation of type I interferon production	NP_004698	[16]

Putative Atg8 homologs	Increases cell-surface expression of kappa-type opioid receptor. Intra-Golgi traffic and transport. Intracellular transport of GABA (A) receptors. Apoptosis. Formation of autophagosomes Formation of autophagosomes. Formation of autophagosomes. Formation of autophagosomes	CAG38511	
GABARAPL1 (Atg8L)		NP_009266.7	
GABARAPL2 (Atg8C)		NP_00209.1	
GABARAP (Atg8A)		NP_07379.1	
MAP1LC3C (Atg8E)		NP_1159031	
MAP1LC3A (Atg8F)		NP_001078950	
MAP1LC3B			
MAP1LC3B2			

Ufm1, UFM1	Protein Ufmylation	NP_057701	[17]

DWNN	Unknown but may be involved in protein ubiquitination involved in ubiquitin-dependent protein catabolic process	2C7H_A	[18]

URM	Unknown	CAI13492	[19]

**Table 2:** Proteins with ubiquitin-like domains.

Protein	Function	Accession Number	Reference
Parkin	Acts as a positive regulator of autophagy. Promotes the autophagic degradation of dysfunctional depolarized mitochondria. Regulates neuron death. Limits the production of reactive oxygen species. Regulates cyclin-E during neuronal apoptosis. May represent a tumor suppressor gene	NP_004553	[108]
UHRF1	Important for G1/S transition. May be involved in DNA repair and chromosomal stability	NP_037414.3	[109]
UHRF2	Important for G1/S transition. May be involved in DNA repair and chromosomal stability	CAI13295.1	[109]
RAD23	Involved in nucleotide excision repair	NP_005044.1	[110]
BAG1	Regulation of proteasomal and lysosomal protein	NP_001165886	[111]
BAT3	Involved in DNA damage-induced apoptosis. Immuno-proteasomes to generate antigenic peptides via targeted degradation, Post-translational delivery of tail-anchored (TA) membrane proteins to the endoplasmic reticulum membrane	BAB63390.1	[112]
DDI1	Protein degradation	NP_001001711.1	[113]
DDI2	Protein degradation	NP_115717.3	[113]
OASL	Immune response cytokine-mediated signaling pathway interferon-gamma-mediated signaling pathway type I interferon-mediated signaling pathway	NP_003724	[114]
HERPUD1	Cellular calcium ion homeostasis. Response to unfolded protein. Endoplasmic reticulum unfolded protein response. Negative regulation of caspase activity	NP_001010989	[115]
RBBP6	Unknown but may be involved in protein ubiquitination involved in ubiquitin-dependent protein catabolic process	NP_008841.2	[104]
UBQLN1 (Ubiquilin)	Response to Hypoxia. Apoptosis. Regulation of ubiquitination	NP_038466	[116]

**Table 3:** Drugs that target the Ubiquitin-proteasome system and Ubls.

Drug	Mode of Action	Target	Disease	State of Development	Reference
Bortezomib/Velcade^®^	Selective proteasome inhibitor	Proteasome	Multiple myeloma	In clinical use	[135–137]
Nutlin	Mdm2 inhibitor	Mdm2	Multiple myeloma	Preclinical	[141]
Multiple approaches to target SUMO pathway	Inhibition of the active site Inhibiting association with E1 Blocking association with target protein	The E2 enzyme in the SUMO pathway UBC9	Cancer	Experimental phases	[146]
Arsenic trioxide	Targets SUMOylation	Degradation of PML–RAR-α	Leukemia	In use clinically	[146]
All-trans retinoic acid	See above	See above	See above	See above	[146]
MLN-4924	Inhibits NEDD8 E1-activating enzyme (NAE)	NEDD8 activating enzyme (NAE)	Cancer, Multiple myeloma and Hodgkin’s lymphoma	Phase II clinical trials	[147] [148]
HBX 41108	Inhibits USP7 deubiquitinating activity	Ubiquitin-specific proteases (USP)	Cancer	Phase I	[149]
JNJ26854165	Inhibitor	E3-Hdm2	Multiple myeloma and solid tumors	Phase I	[148]
GDC-0152	Inhibitor	E3-IAP	Metastatic malignancies	Phase I	[148]
LCL161	Inhibitor	E3-IAP	Solid tumors	Phase I	[148]
AT-406	Inhibitor	E3-IAP	Solid tumors and lymphoma	Phase I	[148]
AEG 35156	Inhibitor	E3-IAP	AML and liver cancer	Phase II	[148]
AEG 40826	Inhibitor	E3-IAP	Lymphoid tumors	Phase I	[148]
TL 32711	Inhibitor	E3-IAP	Solid tumors and lymphoma	Phase I	[148]
YM155	Inhibitor	E3-IAP	Lung cancer	Phase II	[148]
